# The OCTO‐Plus Intervention to Support Families of Chronically Critically Ill Children in Paediatric Critical Care: A Pilot Study

**DOI:** 10.1111/nicc.70294

**Published:** 2025-12-14

**Authors:** Mark T. Marston, Maria‐Helena Perez, Anne‐Sylvie Ramelet

**Affiliations:** ^1^ Institute of Higher Education and Research in Healthcare University of Lausanne Lausanne Switzerland; ^2^ Research Lab on Child and Family Health La Source School of Nursing, HES‐SO Lausanne Switzerland; ^3^ Paediatric Intensive and Intermediate Care Unit, Women‐Mother‐Child Department University Hospital Lausanne Lausanne Switzerland

**Keywords:** family functioning, family nursing, family‐centred care, feasibility studies, intermediate care facilities, paediatric intensive care

## Abstract

**Background:**

Families of chronically critically ill (CCI) children admitted to paediatric critical care (PCC) experience significant and long‐lasting psychological distress. Structured family‐centred care consultations, using a family system approach, have shown promise in mitigating these effects. Though most studies in PCC are single‐component or lack methodological rigour. There is a need for complex, multi‐component interventions that address the evolving needs of families in PCC.

**Aim:**

To assess the feasibility of a co‐developed complex multi‐component family‐centred intervention to support families of CCI children on the PCC ward before effectiveness testing in a future clinical trial.

**Study Design:**

This single‐centre pilot feasibility study used a descriptive observational design guided by Bowen's feasibility framework. The intervention consisted of structured family consultations delivered by trained healthcare professionals, supported by interprofessional handovers. Quantitative data were collected at multiple time points using validated instruments to assess feasibility and limited efficacy outcomes.

**Results:**

Of 802 patients screened, 55 were eligible and 19 families enrolled (34.5%) representing 19 children and 27 parents. All allocated parents completed the intervention. Parents reported clinically meaningful reductions in acute stress, anxiety and depression from admission to follow‐up. Family functioning improved across most domains, and no parent met the threshold for PTSD at follow‐up. Satisfaction scores remained high throughout.

**Conclusions:**

This study demonstrates the clinical feasibility of a multi‐component family‐centred intervention for parents of children hospitalised in paediatric critical care. Findings suggest a protective effect on parental mental health and improved family functioning, supporting progression to a full‐scale effectiveness trial.

**Relevance to Clinical Practice:**

The OCTO‐Plus intervention offers a structured, family‐centred approach to support parents of CCI children in PCC. Its integration into clinical practice may enhance emotional regulation, communication and resilience, ultimately improving long‐term outcomes for families.

## Impact Statements

- What is known about the topic
  ○ Chronic critically ill children in paediatric critical care represent one of the most vulnerable patient populations.
  ○ Families of chronic critically ill children experience profound and sustained stress and require targeted support.
  ○ Family consultations have shown clear benefits in adult critical care and general paediatric settings, improving communication, emotional well‐being and decision‐making.
- What this paper adds
  ○ Introduces a customised, multi‐component family‐centred support intervention tailored to the needs of families of chronic critically ill children.
  ○ Provides a preliminary evaluation of the feasibility of this intervention, offering insights into its potential efficacy in improving family support and care outcomes.
  ○ Provides evidence to justify further testing in a full scale clinical trial to confirm the benefits of such intervention for families.

## 1 Introduction

### 1.1 Background

The hospitalisation of a child in Paediatric Critical Care (*PCC*)—which encompasses both intensive and intermediate care settings—can be an extremely stressful experience for parents and families, often resulting in significant psychological distress. Mental health issues such as anxiety, depression and acute stress disorder are common among these families, with 33%–63% experiencing moderate to severe symptoms [1, 2, 3, 4]. These symptoms are increasingly recognised as components of Post‐Intensive Care Syndrome‐Family (*PICS‐F*), which encompasses psychological, social and functional impairments in family members following a child's critical illness [5, 6]. This distress is particularly pronounced in families of chronically critically ill (*CCI*) children, who endure prolonged and intense medical care [7, 8].

CCI children are typically defined as those requiring prolonged PCC stays due to pre‐existing or acquired co‐morbidities, multi‐organ dysfunction or technology dependence [9, 10]. Representing approximately 10% of PICU admissions, they account for up to 40% of bed occupancy and 80% of total PICU costs, and their care requires complex coordination, continuity and specialised expertise, placing a disproportionate burden on healthcare systems [11, 12, 13, 14].

There is overwhelming evidence showing the negative psychosocial impact of a prolonged PCC stay not only on the patient but also the family. Parents and families experience high stress levels triggered by the child's appearance, uncertainty surrounding diagnostics and outcomes and changes in parental roles [7, 8, 15]. This is especially true for families of CCI children, due to their higher clinical vulnerability and risk of mortality and longer PICU stay, compared to non‐CCI children [16]. Parents with lower education, comorbidities, prior trauma, psychological conditions and impaired family functioning are particularly at risk of experiencing acute stress [1, 17, 18]. Between 35% and 64% of parents experience acute stress within 3 months of admission, and 10%–48% develop depressive symptoms and post‐traumatic stress disorder (*PTSD*) [18, 19, 20, 21, 22, 23]. Parents in PCC, regardless of whether their child is CCI or not, experience anxiety and depression and these symptoms typically peak shortly after PCC admission and may intensify during hospitalisation [2, 24, 25], increasing the risk of long‐term psychological distress [26]. Anxiety and depression, rooted in persistent feelings of powerlessness, often arise from unsuccessful coping strategies [27, 28, 29]. Although CCI parents during a prolonged PCC stay may develop some coping strategies [8], family system approach consultations show some benefits in helping parents develop those strategies early during hospitalisation. Family functioning, encompassing cohesion, communication, coping and resilience, can either buffer or exacerbate PTSD risk [18, 30, 31, 32], and PCC hospitalisation significantly disrupts family functioning, with impairments often lasting months after discharge [8, 18, 33]. During prolonged PCC stays, unmet needs such as communication gaps, care continuity and coordination challenges [34, 35, 36] further destabilise parents and families. Over time, families gradually adapt to the PCC environment and identify key moments for involvement, highlighting the importance of individualised care, active participation and transition preparation [4, 36, 37, 38, 39, 40].

The prevalence of these psychological issues emphasises the urgent need for additional support to what is currently provided as standard family‐centred care. Current interventions building on Family‐Centred Care (*FCC*) support families by increasing their presence in the PICU, enhancing communication and involving them in the decision‐making process [41, 42]. Benefits were shown using structured personalised exchanges, including family therapeutic consultations, to enhance goal setting, shared decision‐making, engagement and family functioning, by emphasising emotional support, cognitive modelling and family empowerment [43]. However, these interventions often lack theoretical foundations leading to heterogeneous outcome measures and a lack of high‐level evidence of effectiveness [44]. In addition, our systematic review shows that interventions promoting parental empowerment lack methodological rigour, and their single‐component nature fails to fully address the complexities of families' needs [45, 46]. The only clustered randomised clinical trial conducted in adult ICU showed a small but significant increase in satisfaction in families who received family support [47]. These results are not directly transferable to the paediatric setting where families have different needs, roles and responsibilities [48]. This warrants the development of a multi‐component intervention. Such interventions are considered complex and, as outlined in the updated Medical Research Council (*MRC*) framework, require careful development and pilot testing to assess feasibility before proceeding to full‐scale effectiveness trials [49]. Assessing the acceptability and feasibility of a co‐developed multi‐component intervention is therefore a critical step towards testing its potential in addressing the multifaceted and evolving needs of CCI children and their families.

### 1.2 Aim

The aim of this pilot study was to assess the feasibility of a co‐developed, complex, multi‐component, family‐centred intervention to support families of CCI children in the PCC ward, prior to effectiveness testing in a future clinical trial.

### 1.3 Research Questions

1. Can family members who would most benefit from an intervention be identified, recruited and kept in the study?
2. Is there preliminary evidence for potential change related to the intervention?

## 2 Design and Methods

### 2.1 Design

This is a single‐centre pilot feasibility study using a descriptive observational design and guided by Bowen's feasibility framework, focusing on indicators for limited efficacy [50] and reported according to CONSORT guidelines for pilot and feasibility trials (see Table S1) [51].

### 2.2 Study Setting and Participants

The study was conducted in a 24‐bed PCC ward, which included 12 intensive care beds and 12 intermediate care beds, within a tertiary referral hospital in Switzerland. We included all parents of CCI children aged between 0 and 17 years with dependence on at least one technology and a length of stay above 8 days [9]. Both parents could be included if they wanted and if so, each parent was considered an individual participant. To be eligible, parents had to be available for at least 2 interviews on day 8 (±48 h) and day 12 (±48 h) during the PCC hospital stay. Participants had to be 18 years of age or above, able to provide written informed consent and understand and speak French.

Parents of children meeting the following criteria were excluded: < 37 gestational weeks, discharge expected within 24 h, transfer from another Swiss PCC ward, withdrawal or withholding treatments or end‐of‐life situations.

### 2.3 Intervention

Based on the results of the previous OCTOPUS studies [3, 8, 16, 52], the *OCTO‐Plus intervention*, a multi‐component family support intervention was developed in collaboration with healthcare professionals and partner parents. The detailed description of this co‐development is described elsewhere (manuscript under review). It consisted of family consultations delivered by 11 trained healthcare professionals (8 expert nurses specialised in intensive care and 3 physicians with an interest in family‐centred care) from the PCC staff. The training consisted of on‐demand online education material, two half‐day hands‐on workshops to practice family interview techniques and an on‐site in‐practice coaching session. PCC healthcare professionals received brief information on FCC and the intervention during two team meetings prior to intervention delivery. Family consultations were conducted with the parents weekly, starting from day 8 until 1 month after PCC discharge. For each family, one or both parents, took part in at least two meetings, at day 8 and at discharge from PCC. Family consultations were semi‐structured interviews grounded in family systems theory [53] and family‐centred care [41]. They provided a comprehensive family systems assessment, emotional support, individualised information and strengthened the parental role. Before and after each family consultation, an interprofessional handover of information related to family needs took place to ensure coherent information, care coordination and follow‐up care.

### 2.4 Data Collection

We collected sociodemographics and clinical characteristics, as well as limited efficacy data from parents of CCI children at various points: on D8 of hospitalisation, during weekly family meetings, at discharge, and 1 month after discharge (Table S2). A sample size of 30 parents is suggested to be satisfactory for feasibility studies and to answer the research questions [54]. Data collection took place from September 2023 to August 2024, using the secured web‐based REDCap software. Quantitative data collected during family interviews through author‐developed checklists was securely stored on the REDCap platform.

Limited efficacy, as a feasibility indicator according to Bowenet al. [50] was estimated in relation to relevant child and parent clinical outcomes. These included family functioning, parental anxiety and depression, parental satisfaction, parental post‐traumatic stress disorder, parental acute stress, child quality of life and child health and functional status (see Table S2). All original instruments used for assessment had moderate to strong psychometric properties and have been translated into French and further validated.

Family functioning was measured using the 36 items, across eight domains, PedsQL‐FIM. Scores are attributed on a 5‐point Likert scale and transformed to a 0–100 scale, higher scores indicating better functioning [55, 56].

Emotional distress—anxiety and depression—in caregivers was assessed using the 4‐item PHQ‐4. Items are rated on a 4‐point scale, with higher scores indicating greater symptom severity [57, 58].

Parent satisfaction was measured using the 30‐item EMPATHIC‐30. Items are rated on a 5‐point Likert scale, with higher scores reflecting greater satisfaction [59, 60].

Acute stress symptoms were measured using the ASDS. Items are rated by symptom frequency, with higher scores indicating more severe acute stress [61].

PTSD symptoms were assessed using the 20‐item PCL‐5. Items are rated on a 5‐point scale, with higher scores indicating greater symptom severity [62, 63].

Child health‐related quality of life was measured using the 23‐item PedsQL. Items are rated on a 5‐point scale and transformed to a 0–100 scale, with higher scores indicating better quality of life [64, 65, 66].

Overall health status was assessed using the POPC scale. It uses a 6‐level ordinal scale, with lower scores indicating better functioning [67].

Functional status was measured using the 6‐domain FSS. Each domain is scored from 1 to 5, with total scores ranging from 6 to 30; higher scores indicate worse functional status [68].

### 2.5 Data Analysis

Analysis of outcomes primarily focused on descriptive statistics, including measures of distribution (mean and standard deviation or median and confidence interval/interquartile range). To identify changes in outcomes, pre‐ and post‐program comparisons were performed using paired *t*‐tests for parametric data and Wilcoxon rank sum tests for non‐parametric data. Trends and potential effects on clinical outcomes were illustrated with spaghetti plots.

### 2.6 Ethical and Institutional Approvals

Ethics approval was obtained from the Cantonal Ethics Commission for human research (CER‐VD) (no. 2023‐00998) on 25 September 2023.

## 3 Results

**FIGURE 1:**
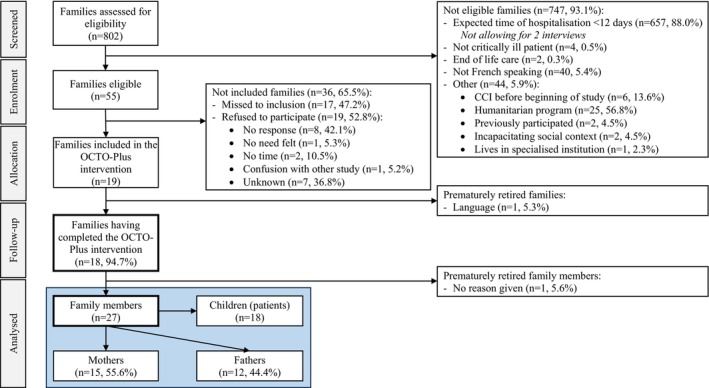
Participants flowchart [51].

We screened a total of 802 patients, of which 55 (6.9%) were eligible. Ultimately, 19 patients and their families were enrolled (34.5%). However, 1 parent (5.3%) withdrew due to the language barrier and availability challenges, and 18 families were allocated (94.7%). All allocated families completed the OCTO‐Plus intervention, despite one parent retiring without reason (5.6%). Of the families not included, 19 (52.8%) refused participation and 17 (47.2%) were missed for inclusion due to resource constraints within the core team—time constraints, workload or unavailability. A detailed overview of recruitment is presented in Figure 1.

### 3.1 Socio‐Demographic and Clinical Characteristics

A total of 18 children and 27 parents were included, representing 18 families. On average, each family had 2.2 (±1.2) children, including the hospitalised child. All included children spoke French, and only 5 (27.8%) were of school age, 4 (22.2%) went to kindergarten, while the remaining 9 (50.0%) were at home due to their age (see Table 1). Out of the 18 CCI children, 10 were girls (55.6%). The mean age of CCI children in PCC was 3.2 years (±4.0), which was not significantly higher than that of patients included, 2.1 years (±2.4, *p* = 0.094). The average length of stay for CCI children included was 22.8 ± 10.9 days and was not significantly lower than that of CCI children not included in the study, staying 28.7 ± 34.3 days on average (*p* = 0.346). Despite having a higher proportion of females within CCI children included, this did not significantly differentiate from the proportion of females within the overall CCI children population (45.9%, *p* = 0.703). All patients had a dependence on at least two technologies except for two children who depended on one technology only. The most frequent technologies were nasogastric tube (*n* = 17, 94.4%), continuous venous infusion (*n* = 14, 77.8%) and invasive and non‐invasive ventilation (*n* = 14, 77.8%). One patient (5.6%) required seven technologies, six patients (33.3%) four, and all others (61.1%) had between one and three.

**TABLE 1:** Socio‐demographic and clinical characteristics of children.

Variables	Levels, values		CCI	CCI	*p*
Items			Included	Not included	
Socio‐demographic characteristics			*N* = 18	*N* = 37	
Age (average)	Years	Mean ± SD	2.1 ± 2.4	3.7 ± 4.5	0.094
Age (categories)	< 12 months	*N* (%)	10 (55.6)		
	12–24 months		4 (22.2)		
	2–4 years		2 (11.1)		
	5–7 years		2 (11.1)		
	8–12 years		0		
	≥ 13 years		0		
	Missing		0		
Gender	Female	*N* (%)	10 (55.6)	17 (45.9)	0.703
	Male		8 (44.4)		
	Missing		0		
Number of children in family	*N*	Mean ± SD	2.2 ± 1.2		
	(1, 2, 3, 4, 5)		(6, 6, 4, 1, 1)		
	Missing		0		
Primary language spoken^a^	French	*N* (%)	18 (100.0)		
	German		0		
	Italian		1 (5.6)		
	Spanish		1 (5.6)		
	Portuguese		0		
	Other (1 Romanian, 1 Serbo‐Croatian, 1 Sign language, 1 no speech)		4 (22.2)		
			0		
	Missing				
Child education	Primary or secondary school	*N* (%)	5 (27.8)		
	High school		0		
	Traineeship		0		
	Other (4 Kindergarten, 6 at home, 3 na)		13 (72.2)		
	Missing		0		
Length of stay^b^	Days	Mean ± SD	22.8 ± 10.9	28.7 ± 34.3	0.346

Variables (instruments)	Levels, values		t1	fu	Difference
Domains, items					(fu − t1)
Technology dependence			*N* = 18		
Tracheostomy	Technology present: 1 = Yes 0 = No	*N* (%)	0		
Invasive and non‐invasive ventilation			14 (77.8)		
Nasogastric or jugenal tube			17 (94.4)		
Inotropic medication			1 (5.6)		
Analgosedative medication			8 (44.4)		
Continuous intravenous infusion			14 (77.8)		
Ventriculo‐peritoneal derivation			0		
Parenteral nutrition			1 (5.6)		
Hemo‐ or peritoneal dialysis			0		
ECMO			1 (5.6)		
Berlin heart			0		
Health status (POPC)			*N* = 18	*N* = 16	∆ ± SE
Overall health status	Currently: 1 = Good health status 2 = Light disability 3 = Moderate disability 4 = Severe disability 5 = Vegetative state or coma	Mean ± SD	2.17 ± 1.29	1.56 ± 0.89	−0.60 ± 0.38
Functional status (FSS)			*N* = 18	*N* = 14	∆ ± SE
Mental status	Currently: 1 = No dysfunction (normal) 2 = Mild dysfunction 3 = Moderate dysfunction 4 = Severe dysfunction 5 = Very severe dysfunction	Mean ± SD	1.44 ± 0.98	1.00 ± 0.00	−0.44 ± 0.23***
Sensory functioning			1.06 ± 0.24	1.00 ± 0.00	−0.06 ± 0.06
Communication			1.78 ± 1.31	1.07 ± 0.27	−0.71 ± 0.32*
Motor functioning			1.39 ± 0.92	1.00 ± 0.00	−0.39 ± 0.22
Feeding			2.33 ± 1.14	1.64 ± 1.08	−0.69 ± 0.39
Respiratory status			2.50 ± 1.69	1.14 ± 0.54	−1.36 ± 0.42*
Total score			10.50 ± 4.51	6.86 ± 1.56	−3.64 ± 1.14*
Overall child QOL, parent reported (author developed)				*N* = 13	
Child quality of life	Currently: 0 = Minimal QOL 10 = Maximum QOL	Mean ± SD		7.96 ± 1.76	
Child QOL, parent reported (PedsQL)			2–4, *N* = 3; 5–7, *N* = 3		
A. Physical function	Domain impacted in last month: 100 = Never 75 = Almost never 50 = Sometimes 25 = Frequently 0 = Almost always	Mean ± SD	2–4: 97.9 ± 3.61 5–7: 37.5 ± 54.5		
B. Emotional state			2–4: 73.3 ± 5.77 5–7: 73.3 ± 20.8		
C. Relation to others			2–4: 85.0 ± 26.0 5–7: 81.7 ± 18.9		
D. School			2–4: 33.3 ± na (*N* = 1) 5–7: 51.7 ± 38.2		

*Note:* t1 = day 8, fu = at follow‐up; Significance level: *p < 0.05, **p < 0.01, ***p < 0.001.

Abbreviations: SD, standard deviation; SE, standard error.

^a^ Parametric *t*‐test.

^b^ One major outlier (male, age = 5.5 years, IMCU LOS = 832 days) was removed from analysis involving LOS because disproportionately skewing results and limiting interpretation.

The parents' sample included 15 mothers (53.6%) and 12 fathers (46.4%). Most parents were between 30 and 39 years old (*n* = 24, 74.1%) (see Table 2). Most parents were either married or in a registered partnership (*n* = 18, 66.7%). All families were fluent in French, and 16 (59.3%) were Swiss nationals. Half were Christians (*n* = 14, 51.9%) and 8 (29.6%) had no religious affiliation. Twelve parents (44.4%) had completed an apprenticeship and 11 (40.7%) had higher education or a university degree. Some were on parent leave (*n* = 7, 25.9%), but most remained active during hospitalisation (*n* = 19, 70.4%), either working independently or being employed. Just under half the parents (12, 44.4%) reported an annual income of CHF 50001–100 000 (approximately 44 000–88 000 GBP), and three parents (12.5%) declared a higher income. Almost one‐third of the parents (*n* = 7, 25.9%) indicated that their financial situation was problematic.

**TABLE 2:** Socio‐demographic and clinical characteristics of parents.

Variables items	Levels, values		Parents included
Socio‐demographic and clinical characteristics			*N* = 27
Age	18–29 years	*N* (%)	3 (11.1)
	30–39 years		20 (74.1)
	40–49 years		4 (14.8)
	50–59 years		0
	> 60 years		0
	Missing		0
Gender	Female	*N* (%)	15 (55.6)
	Male		12 (44.4)
	Missing		0
Marital status	Married/registered partnership	*N* (%)	18 (66.7)
	Divorced/seperated		2 (7.4)
	Single		7 (25.9)
	Widowed		0
	Missing		0
Nationality	Swiss	*N* (%)	16 (59.3)
	French		3 (11.1)
	German		1 (3.7)
	Italian		0
	Spanish		0
	Portuguese		1 (3.7)
	Other		6 (22.2)
	2 Belgium, 1 Romanian		0
	1 Tunisian, 1 Venezuelan, 1 na		
	Missing		
Primary language spoken^a^	French	*N* (%)	27 (100.0)
	German		3 (11.1)
	Italian		4 (14.8)
	Spanish		1 (3.7)
	Portuguese		0
	Other		7 (25.9)
	5 English, 1 English‐Romanian, 1 Serbo‐Croatian		0
	Missing		
Religious affiliation	Christianity (protestant/catholic)	*N* (%)	1 (3.7)/13 (48.1)
	Islam		3 (11.1)
	Hinduism		0
	Judaism		0
	None		8 (29.6)
	Other		2 (7.4)
	2 Orthodox		0
	Missing		
Education	No diploma	*N* (%)	1 (3.7)
	Boarding school		3 (11.1)
	High‐school		0
	Apprenticeship		12 (44.4)
	Higher education		5 (18.5)
	University		6 (22.2)
	Missing		0
Occupation	Independent activity	*N* (%)	4 (14.8)
	Employed		15 (55.6)
	Studying		0
	Unemployed		0
	No paid activity		0
	Sick leave		1 (3.7)
	Mother/fatherhood leave		7 (25.9)
	Unpaid leave		0
	Retired		0
	Missing		0
Personal income	< 50 000	*N* (%)	8 (33.3)
	50 001–100 000		12 (50.0)
	100′001–150′000		3 (12.5)
	150 001–200 000		0
	> 200 000		1 (4.2)
	Missing		3 (11.1)
Problematic financial situation	Yes	*N* (%)	7 (25.9)
	Missing		0
Time from home to critical care unit	< 30 min	*N* (%)	6 (22.2)
	30–60 min		13 (48.1)
	> 60 min		8 (29.6)
	Missing		0
Overnight sleeping provided by hospital	Yes	*N* (%)	11 (40.7)
	Missing		0
Time spent at bedside in critical care	Hours per week	Mean ± SD	65.6 ± 32.5
	Missing		0
Physical health	Bad	*N* (%)	0
	Moderate		4 (14.8)
	Good		9 (33.3)
	Very good		8 (29.6)
	Excellent		6 (22.2)
	Missing		0
Person available for information (friend/family)	Average	Mean ± SD	8.2 ± 8.2
	0	*N* (%)	1 (3.7)
	1		1 (3.7)
	2		2 (7.4)
	3		4 (14.8)
	4		4 (14.8)
	5		3 (11.1)
	8		1 (3.7)
	10		7 (25.9)
	15		1 (3.7)
	20		2 (7.4)
	40		1 (3.7)
	Missing		0
Person available for help (friend/family)	Average	Mean ± SD	8.2 ± 7.2
	0	*N* (%)	1 (3.7)
	1		1 (3.7)
	2		4 (14.8)
	3		2 (7.4)
	4		4 (14.8)
	5		2 (7.4)
	10		9 (33.3)
	20		3 (11.1)
	30		1 (3.7)
	Missing		0
Type of person available^a^	Family	*N* (%)	25 (92.6)
	Distant relative		3 (11.1)
	Friend		20 (74.1)
	Neighbour		7 (25.9)
	Colleague		9 (33.3)
	Other		0
	Missing		0

^a^ Multiple answers possible.

Parents generally took less than 1 h to travel from their home to the hospital (19, 70.3%), and eight (29.6%) travelled a greater distance. Overnight accommodation near the hospital was provided to 11 parents (40.7%). On average, parents spent 69.0 h (±31.7) a week at their child's bedside in PCC. Notably, a relevant difference was observed between mothers and fathers, with mothers spending an average of 76.1 h (±28.9) a week compared to fathers, who spent 49.6 h (±26.7) a week at the bedside (*p* = 0.060). While some parents reported a moderate impact on their physical health (4, 14.8%), the majority described their physical health as good to excellent (*n* = 23, 85.2%). When seeking support, parents relied on a similar number of people for information (*n* = 8.2 ± 8.2) and assistance (*n* = 8.2 ± 7.2). However, there was considerable variation in the number of contacts. Most parents relied on five or fewer individuals for information (*n* = 15, 55.6%) and for help (*n* = 14, 51.9%). Some parents counted as many as 20 to 40 individuals for information (*n* = 3, 11.1%) and for help (*n* = 4, 14.8%). Most frequently, these individuals were family members and close relatives (92.6%; 25 individuals) or friends (74.1%; 20 individuals).

### 3.2 Limited‐Efficacy Endpoints

Acute stress scores peaked 2–3 weeks after PCC admission (62.7 ± 31.8), decreased at discharge (46.9 ± 16.9) and further at follow‐up (38.6 ± 14.1) (see Table 3). The mean overall symptom severity PTSD score was 14.2 ± 13.9 at follow‐up and no family met the threshold for PTSD. Overall anxiety and depression symptoms at admission had an average score of 6.6 ± 3.6, which decreased at discharge (3.7 ± 3.6) and further declined at follow‐up (2.8 ± 3.7). Family functioning scores improved from 57.6 ± 18.2 on admission to 61.7 ± 20.9 at discharge and 69.0 ± 21.6 at follow‐up. Spaghetti plots highlight the trend of positive changes across all PedsQL‐FIM subscales, except for *Cognitive functioning* (see Figure S1).

**TABLE 3:** Limited efficacy endpoints.

Variables (instruments)	Levels, values	t1	Td	Difference (td − t1)	fu	Difference (fu − t1)
		Mean ± SD	Mean ± SD	∆ ± SE	Mean ± SD	∆ ± SE
Domains (items)						
Anxiety and depression (PHQ‐4)		N = 27	N = 21		N = 18	
Anxiety (2)	Symptom occurrence in last 2 weeks: 0 = Never 1 = Several days 2 ≥ days 3 = Almost every day	3.56 ± 1.78	2.15 ± 1.99	−1.41 ± 0.52***	1.67 ± 2.00	−1.89 ± 0.58*
Depression (2)		3.04 ± 2.14	1.59 ± 1.80	−1.44 ± 0.54	1.17 ± 1.92	−1.87 ± 0.61**
Overall (4)		6.59 ± 3.64	3.74 ± 3.63	−2.85 ± 0.99**	2.83 ± 3.65	−3.76 ± 1.11***
Satisfaction (EMPATHIC‐30)		N = 27	N = 21		N = 18	
A. Information (5)	Item occurrence since admission: 1 = Certainly no 2 = No 3 = Somewhat/probably no 4 = Somewhat/probably yes 5 = Yes 6 = Certainly yes	5.31 ± 0.61	5.37 ± 0.58	+0.05 ± 0.17	5.44 ± 0.57	+0.13 ± 0.18
B. Care and cure (8)		5.64 ± 0.53	5.56 ± 0.45	−0.07 ± 0.14	5.57 ± 0.51	−0.06 ± 0.16
C. Organisation (5)		5.25 ± 0.80	5.18 ± 0.63	−0.07 ± 0.21	5.49 ± 0.51	+0.24 ± 0.20**
D. Parental participation (6)		5.32 ± 0.63	5.37 ± 0.55	+0.04 ± 0.17	5.54 ± 0.47	+0.22 ± 0.16
E. Professional attitude (6)		5.61 ± 0.66	5.74 ± 0.37	+0.13 ± 0.15**	5.72 ± 0.38	+0.11 ± 1.56
Satisfaction total items (30)		5.43 ± 0.56	5.44 ± 0.42	+0.02 ± 0.14	5.55 ± 0.41	+0.13 ± 0.14*
Family functioning (PedsQL‐FIM)		N = 27	N = 21		N = 17	
A. Physical functioning (6)	Domain impacted in last month: 100 = Never 75 = Almost never 50 = Sometimes 25 = Frequently 0 = Almost always	43.5 ± 19.0	50.2 ± 25.4	+6.68 ± 6.64	59.6 ± 26.3	+16.00 ± 7.35
B. Emotional functioning (5)		50.7 ± 24.7	59.5 ± 28.3	+8.78 ± 7.79	70.6 ± 26.0	+19.80 ± 7.89***
C. Social functioning (4)		56.0 ± 23.0	63.4 ± 26.2	+7.37 ± 7.23	71.7 ± 24.6	+15.70* ± 7.44
D. Cognitive functioning (5)		72.0 ± 23.7	66.2 ± 23.7	−5.85 ± 6.90	70.0 ± 24.7	−2.04 ± 7.53
E. Communication (3)		70.4 ± 26.4	73.8 ± 27.8	+3.44 ± 7.91	83.8 ± 25.6	+13.50 ± 8.02**
F. Worry (5)		47.4 ± 23.6	55.2 ± 24.9	+7.83 ± 7.08	57.4 ± 28.6	+9.95 ± 8.30
G. Daily activities (3)		38.0 ± 31.6	43.7 ± 35.1	+5.69 ± 9.79	59.3 ± 29.0	21.40* ± 9.30
H. Family relationships (5)		82.8 ± 19.2	82.1 ± 21.0	−0.64 ± 5.88	84.1 ± 21.2	+1.34 ± 6.33
Parent QOL summary (20, A‐D)		55.0 ± 18.3	59.2 ± 23.2	+4.21 ± 6.15	67.4 ± 22.8	+12.4 ± 6.55*
Family QOL summary (8, G‐H)		66.0 ± 20.7	67.7 ± 22.9	+1.74 ± 6.39**	74.8 ± 22.6	+8.84 ± 6.77
Total QOL impact score (36, A‐H)		57.6 ± 18.2	61.7 ± 20.9	+4.10 ± 5.75	69.0 ± 21.6	+11.40 ± 6.29*
Post‐traumatic stress, PTSD (PCL‐5)					N = 18	
A. Intrusion (5)	Symptoms occurrence/impact in last month: 0 = Not at all 1 = A little 2 = Medium 3 = Frequently 4 = Extremely				4.28 ± 5.49	
B. Avoidance (2)					1.28 ± 2.02	
C. Cognition and mood (7)					3.67 ± 4.10	
D. Arousal (6)					4.94 ± 4.63	
Overall symptom severity (20)					14.2 ± 13.9	

		t1	t2	Difference (t2 − t1)	t3	Difference (t3 − t1)	t4	Difference (t4 − t1)	td	Difference (td − t1)	fu	Difference (fu‐t1)
Variable (instrument) Domains	Levels, values	Mean ± SD	Mean ± SD	∆ ± SE	Mean ± SD	∆ ± SE	Mean ± SD	∆ ± SE	Mean ± SD	∆ ± SE	Mean ± SD	∆ ± SE
Acute stress (ASDS)		N = 25	N = 7		N = 3		N = 1		N = 17		N = 20	
1. Dissociative (5)	Symptom occurrence: 1 = Not at all 5 = Extremely	11.30 ± 5.28	12.40 ± 6.45	+1.15 ± 2.66	16.30 ± 8.08	+5.05 ± 4.78	6.00 ± na	−5.28 ± na	11.10 ± 5.06	−0.16 ± 1.62*	8.50 ± 4.08	−2.78 ± 1.40*
2. Re‐experiencing (4)		9.84 ± 4.42	11.70 ± 5.38	+1.87 ± 2.22	14.70 ± 9.24	+4.83 ± 5.41	4.00 ± na	−5.84 ± na	10.30 ± 5.22	+0.45 ± 1.54	8.80 ± 3.72	−1.04 ± 1.21
3. Avoidance (4)		8.52 ± 3.64	8.71 ± 3.45	0.19 ± 1.49	9.33 ± 1.15	+0.81 ± 0.99*	4.00 ± na	−4.52 ± na	8.65 ± 5.09	+0.13 ± 1.43	7.50 ± 4.32	−1.02 ± 1.21
4. Arousal (6)		15.80 ± 6.92	15.00 ± 6.16	−0.80 ± 2.71	22.30 ± 13.30	+6.53 ± 7.79	9.00 ± na	−6.80 ± na	16.90 ± 7.04	+1.08 ± 2.20	13.80 ± 5.41	−2.00 ± 1.84*
Total score		45.40 ± 16.50	47.90 ± 19.60	+2.42 ± 8.13	62.70 ± 31.80	+17.20 ± 18.60	23.00 ± na	−22.4 ± na	46.90 ± 16.90	+1.50 ± 5.26	38.60 ± 14.10	−6.84 ± 4.57

*Note:* t1 = day 8, t2 = day 12, t3–t4 = weekly, td = at discharge, fu = at follow‐up. Significance level: *p < 0.05, **p < 0.01, ***p < 0.001.

Due to the low number of older children, only three parents were able to provide quality‐of‐life (*QOL*) scores for children aged 2–4 years (pre‐school) and 5–7 years (school‐age). The lowest‐scoring QOL domain for 2‐ to 4‐year‐old children was *School* (33.3 ± na) and the highest was *Physical function* (97.9 ± 3.6). For children aged 5–7 years, the lowest scoring QOL domain was *Physical function* (37.5 ± 54.5), closely followed by *School* (51.7 ± 38.2) and the highest was *Relation to others* (81.7 ± 18.9).

Parent satisfaction scores varied with averages ranging between 5.31 ± 0.61 and 5.64 ± 0.53 at admission, between 5.18 ± 0.63 and 5.74 ± 0.37 at discharge and between 5.44 ± 0.57 and 5.72 ± 0.38 at follow‐up. The domain scoring lowest was *Organisation* and the highest was *Professional attitude*.

## 4 Discussion

This is the first study to our knowledge to pilot test a newly developed complex multi‐component family‐centred intervention to support families of CCI children in PCC. Bowen offered a feasibility framework covering relevant and meaningful dimensions to answer our research questions [69].

Socio‐demographic and clinical characteristics of included patients and parents were comparable to the overall CCI population in the PICU described in another study [16]. The sample was well balanced in terms of gender, employment status and existing social support (friends and family), enhancing the representativeness of findings. Recruitment of families for this study was challenging with low but usual participation rates (34.5%). Paediatric research is typically confronted with ethical concerns related to the population's vulnerability, the emotional burden on families and the consideration of complex care needs within a highly stressful environment [70]. In our study, several adjustments were made to increase recruitment rates: changing the wording from an *intervention* to a *family support program*, highlighting the importance of addressing parents' concerns and offering higher flexibility in arranging therapeutic family conversations. These measures had a significant impact, increasing participation rates by 60% after their introduction. The use of appropriate and accessible language and assurance of a protective environment to conduct family conversations also helped retain a high level of participation in follow‐up meetings. Overall, the recruitment rates—allocation and follow‐up—are encouraging compared to rates reported in other similar studies [71]. We also acknowledge a potential risk of selection bias in our study, as recruitment was limited to French‐speaking parents [72]. However, this bias seems minor, considering the high number of non‐Swiss nationals included.

Our findings support a potential protective effect of the OCTO‐Plus intervention on parental mental health, attested by a clinically meaningful reduction in overall stress levels from admission to follow‐up [20, 73]. The reduction in acute stress both at discharge and 1 month later was most notable in dissociative and re‐experiencing domains, which are core features of PTSD that are particularly responsive to trauma‐focused cognitive behavioural therapy [74]. Such therapies, often embedded in multidisciplinary approaches, address trauma memory processing and emotional regulation, helping reduce intrusive thoughts, such as unwanted memories, nightmares or flashbacks [75]. Central components of the OCTO‐Plus intervention follow such an approach by focusing on grounding techniques (review events, give information, goal‐setting, develop rituals), mindfulness (narrative diary writing, normalising lived experiences) and psychoeducation (active family participation and involvement, parenting skills, acknowledging and emphasising intrinsic strengths) to improve emotional regulation and help recovery by enhancing present‐moment awareness [76, 77]. This strongly supports the targeted effect of integrated psychological support provided by the OCTO‐Plus intervention [78]. We observed low anxiety and depression symptom levels in our sample of parents. Similar results were found in two other studies [20, 79]. This is an important finding because parents with low anxiety and depression symptoms at discharge are less likely to develop PTSD later on [26], further supporting the potential capacity of the OCTO‐Plus intervention to limit long‐term risks.

Family functioning was well improved at discharge and follow‐up compared to 8 days after admission. While the cognitive function and family relationships domains remained largely unchanged, notable improvements were observed in physical, emotional and social functioning, as well as communication, worries and daily activities. These domains align closely with the functional dimension of the Calgary Family Assessment Model, which focuses on how family members interact, communicate and support each other in daily life [53]. The OCTO‐Plus intervention, rooted in this model, emphasises the intrinsic strengths of families, facilitating beliefs and their capacity to adapt and cope with stressors. By fostering emotional regulation, enhancing communication and supporting role distribution within the family, the OCTO‐Plus intervention likely contributed to improved family functioning [80] and sets the basis for improving long‐term resilience [81].

## 5 Limitations

A limitation of this study was the exclusion of patients and siblings from family consultations, focusing instead on parental participation to test intervention feasibility. Several reasons supported this choice: most patients are too young (median age 1.18 years) or sedated to participate and confidentiality would be difficult to ensure in shared hospital spaces [82]. Data from our ongoing PICSS‐PF study (CER‐VD project ID, 2022‐02128) [83] show that a small proportion (7.5%) of siblings of PICU patients participated in the study, making the pertinence and feasibility of their inclusion in our pilot study questionable. Including more participants would have extended the duration of the family consultations beyond the planned 30–45 min and complicated scheduling, especially with siblings' school or childcare commitments [48]. This would also have required highly trained professionals able to conduct interviews with young children, raising concerns about resource demands and sustainability of the intervention. These contextual and methodological factors justified participation centred on parents.

Finally, one limitation inherent to the study design includes the rather small sample size that precludes generalisation of the results to the wider CCI community. Nevertheless, this was not intended in this feasibility study, and further testing of the intervention is warranted.

## 6 Practice Implications and Recommendations for Further Research

The OCTO‐Plus intervention offers a structured, family‐centred approach to support parents of CCI children in PCC. Its integration into clinical practice may enhance emotional regulation, communication and resilience, ultimately improving long‐term outcomes for families.

The estimates in this feasibility study provide an empirically grounded range for a subsequent effectiveness study. Using recruitment rates and variance from this pilot study will help inform sample size calculations for future research, accounting for observed attrition and variability [84].

## 7 Conclusion

This study demonstrates the clinical feasibility of a multi‐component family‐centred intervention for parents of children hospitalised in paediatric critical care. Parents were successfully enrolled in the intervention and their participation could be upheld until follow‐up. This supports the acceptability of the OCTO‐Plus intervention.

The results show a trend of positive change in outcomes and suggest a protective effect on parental mental health and improved family functioning, supporting progression to a full‐scale effectiveness trial.

## Author Contributions

**Mark T. Marston:** writing – review and editing, writing – original draft, visualization, project administration, methodology, investigation, funding acquisition, formal analysis, fata curation, conceptualization. **Anne‐Sylvie Ramelet:** writing – review and editing, visualization, supervision, project administration, methodology, investigation, funding acquisition, formal analysis, data curation, conceptualization. **Maria‐Helena Perez:** writing – review and editing, visualization, supervision, project administration, methodology, investigation, funding acquisition, formal analysis, data curation, conceptualization.

## Funding

A.‐S.R. and M.‐H.P. received a preparatory grant (213921) from the Swiss National Science Foundation to engage with patients and families for the development of the OCTO‐Plus intervention, and as part of an Investigator‐initiated Clinical Trial call.

## Ethics Statement

Ethics approval was obtained from the local human research ethics committee (no. CER‐VD 2023‐000998, on 25/09/2023).

## Consent

Written informed consent was obtained from all included participants.

## Conflicts of Interest

The authors declare no conflicts of interest.

## Supporting information


**Figure S1:** Limited‐efficacy endpoints, graphical presentation.

**Table S1:** CONSORT checklist for pilot and feasibility trials.

**Table S2:** Variables and data collection timepoints.

## Data Availability

The data that support the findings of this study are available from the corresponding author upon reasonable request.
