# Supplementary material for: The OCTO‐Plus Intervention to Support Families of Chronically Critically Ill Children in Paediatric Critical Care: A Pilot Study
Source: Nurs Crit Care. 2025 Dec 14;31(1):e70294. doi: 10.1111/nicc.70294 (PMC12703065; doi:10.1111/nicc.70294)
Supplement: Supplementary file 1 — Figure S1: Limited‐efficacy endpoints, graphical presentation. [file NICC-31-0-s002.docx]

# Figure S1. Limited-efficacy endpoints, graphical presentation.

**Acute stress disorder scale (ASDS) by symptom categories**

**Acute stress score**

**Timepoints**

**Family functioning (PedsQL-FIM) by subscales**

**Timepoints**

**FIM score**
