# Supplementary material for: The OCTO‐Plus Intervention to Support Families of Chronically Critically Ill Children in Paediatric Critical Care: A Pilot Study
Source: Nurs Crit Care. 2025 Dec 14;31(1):e70294. doi: 10.1111/nicc.70294 (PMC12703065; doi:10.1111/nicc.70294)
Supplement: Supplementary file 3 — Table S2: Variables and data collection timepoints. [file NICC-31-0-s001.docx]

# Table S2. Variables and data collection timepoints.

|  | | Baseline assessment^a^ | Post-admission | Admission to discharge | Discharge from PCC | Post-PCC follow-up |
| --- | --- | --- | --- | --- | --- | --- |
| Timepoint | | **t1** | **t2** | **t3-t4** | **td** | **fu** |
| Measurement | | D8 ±48Hrs | D12 ±48Hrs | Weekly ±48Hrs | Discharge ±48Hrs | Discharge +1M^b^ |
| **Parent data (self-perceived)** | | | | | | |
| Parent socio-demographics | | X |  |  | X | X |
| Family functioning | Peds-QL-FIM | X |  |  | X | X |
| Anxiety and depression | PHQ-4 | X |  |  | X | X |
| Satisfaction | EMPATHIC-30 | X |  |  | X | X |
| Post-Traumatic Stress Disorder | PCL-5 |  |  |  |  | X |
| Acute Stress Disorder^c^ | ASDS | X | X | X | X | X |
| **Child data (parent-reported)** | | | | | | |
| Child socio-demographics | | X |  |  |  |  |
| Quality of life | Peds-QL | X |  |  |  | X |
| **Patient health records (researcher retrieved)** | | | | | | |
| Health and functional status | POPC/FSS | X |  |  | X | X |
